# Deficiency of activation-induced cytidine deaminase in a murine model of ulcerative colitis

**DOI:** 10.1371/journal.pone.0239295

**Published:** 2020-09-17

**Authors:** Laura P. Hale

**Affiliations:** Department of Pathology, Duke University Medical Center, Durham, NC, United States of America; Chang Gung University, TAIWAN

## Abstract

Patients with inflammatory bowel disease (IBD) have an increased risk of colorectal cancer, particularly in ulcerative colitis (UC) when the majority of colon epithelial cells may be exposed to inflammation-associated mutagenesis. In addition to mutagenesis generated by oxidative stress, inflammation can induce activation-induced cytidine deaminase (*Aicda*), a mutator enzyme in the APOBEC family, within colon epithelial cells. This study tested the hypothesis that deletion of the *Aicda* gene could protect against the development of inflammation-associated colorectal cancers, using a model of UC-like colitis in “T/I” mice deficient in TNF and IL10. Results showed that T/I mice that were additionally *Aicda*-deficient (“TIA” mice) spontaneously developed moderate to severe UC-like colitis soon after weaning, with histologic features and colon inflammation severity scores similar those in T/I mice. Although the mean survival of TIA mice was decreased compared to T/I mice, multivariable analysis that adjusted for age when neoplasia was ascertained showed a decreased numbers of neoplastic colorectal lesions in TIA mice, with a trend toward decreased incidence of neoplasia. *Aicda* deficiency increased serum IL1α and slightly decreased IL12p40 and M-CSF, as compared with T/I mice, and led to undetectable levels of IgA, IgG1, IgG2a, IgG2b, and IgG3. Taken together, these studies show that *Aicda* deficiency can decrease the number of neoplastic lesions but is not sufficient to prevent the risk of inflammation-associated colorectal neoplasia in the setting of severe UC-like inflammation. The TIA model may also be useful for assessing the roles of antibody class-switch recombination deficiency and somatic hypermutation on regulation of microbiota and inflammation in the small intestine and colon, as well as the pathogenesis of colitis associated with hyper-IgM syndrome in humans. Further studies will be required to determine the mechanisms that drive early mortality in TIA mice.

## Introduction

Cancer is known to result from accumulation of non-lethal mutations and changes in gene regulation that affect cell growth and genomic stability and allow continuous self-renewal. General risk factors for colorectal cancer include older age, family history of colon cancer, smoking, alcohol consumption, being overweight, and not exercising. A personal history of inflammatory bowel disease (IBD) further increases colorectal cancer risk beyond that of the general population, although reported relative risks have varied widely depending on the populations studied [1–4]. Cancer risk in IBD appears to be proportional to the area of colon that is inflamed, with a lower risk in Crohn disease (CD) where inflammation is typically focal and higher in ulcerative colitis (UC) where inflammation is characteristically geographically continuous such that more colon epithelial cells are potentially exposed to inflammation-associated mutagenesis [4]. Although the oxidative stress generated by inflammation within the colon can clearly enhance the rate of mutation accumulation, this alone appears to be insufficient to explain the increased risk of colorectal cancer in IBD patients.

The apolipoprotein B mRNA editing enzyme, catalytic polypeptide-like (APOBEC) family mutator enzyme called activation-induced cytidine deaminase (gene = *Aicda;* protein = AID) is normally expressed during B cell development to facilitate the mutations and DNA rearrangements that enhance antibody diversity [5]. However, a variety of pro-inflammatory cytokines have also been found to induce *Aicda* in colon epithelial cell lines. AID also appears to be highly expressed *in vivo* by colon epithelial cells from humans with IBD [6]. Importantly, forced expression of AID in colon epithelial cells *in vitro* was shown to enhance acquisition of inactivating mutations in the *Tp53* gene after as little as 6 weeks of culture [6]. Unrelated studies showed that AID expression facilitated induction of the *OCT4* and *NANOG* genes that are associated with self-renewal (“stemness”) [7], perhaps via its contribution to methylation/ demethylation reactions that regulate gene expression [8]. Changes in methylation and inappropriate expression of stem cell genes can contribute to the continuous self-renewal that is a hallmark of cancer. Together, these observations suggest that inflammation-induced induction of endogenous *Aicda* in colon epithelium may drive the development of colorectal cancers by enhancing survival and self-renewal, in addition to enhancing mutagenesis & decreasing genomic stability. This study tested the hypothesis that deletion of the *Aicda* gene could protect against the development of inflammation-associated colorectal cancers driven by the above mechanisms.

Testing this hypothesis requires an appropriate animal model. Gene variants for IL-10 and its receptor increase risk for developing both CD [9] and UC in humans [10, 11]. *Il10*^-/-^ mice on the C57BL/6 background readily develop moderate to severe colitis when their mucosal barrier is compromised by exogenous triggers such as infection with Helicobacter species [12, 13] or exposure to non-steroidal anti-inflammatory drugs (NSAIDs) [14, 15]. Colitis in *Il10*^-/-^ mice is characterized by colonic inflammation that is transmural but discontinuous, forming “skip lesions” similar to what is observed in humans with CD. *Il10*^-/-^ mice with colitis also have an increased risk of dysplasia and invasive colon cancer relative to control mice [12, 13, 16–18]. *Aicda* deficiency was previously reported to decrease inflammation-associated colon cancer in mice, based on the observation that adenocarcinoma had developed in 6 of 22 *Il10*^-/-^ mice vs. 1 of 23 *Il10*^-/-^ *Aicda*^-/-^ mice by ~1 year of age (p = 0.05; [19]). However, *Aicda* deficiency did not affect the percentage of mice with precursor dysplastic lesions in that model (90.9% for *Il10*^-/-^ and 90.3% for *Il10*^-/-^ *Aicda*^-/-^ mice) [19]. Furthermore, the percentage of the colonic area involved by inflammation and thus at risk for inflammation-associated neoplasia was not reported in this CD-like model.

We previously reported a novel murine model of colitis that closely resembles human UC rather than CD [20]. *Il10*^-/-^ mice that are also TNF-deficient (*Tnf* ^-/-^ *Il10*^-/-^; termed “T/I” mice) uniformly develop moderate to severe colitis by age 4–6 weeks without the need for exogenous triggering [20]. Inflammation in these mice always involves the rectum and continues proximally in a linear fashion, typically affecting the entire colon (“pancolitis”). The lamina propria of inflamed tissues is packed with inflammatory cells. Crypt abcesses and ulceration are common, but inflammation generally does not extend below the muscularis mucosae. This clinical and histologic pattern closely resembles what is typically seen in human UC. Like humans with UC, T/I mice develop non-polypoid (“flat”) colonic neoplasia with high penetrance (63% incidence by age 28 weeks, with a mean of 2 lesions/mouse) [20]. Based on its extremely close clinical and histologic similarities to human UC, the T/I model may more accurately reproduce mechanisms of inflammation-associated carcinogenesis that are relevant in humans with UC compared with CD-like murine models.

The purpose of this study was to determine how AID presence or absence affected inflammation severity and the incidence of colorectal neoplasia in the T/I mouse model of inflammation-associated colorectal cancer.

## Materials and methods

### Animal studies

C57BL/6 *Tnf* ^-/-^ *Il10*^*-/-*^ (“T/I”) mice were created by crossing founder mice obtained from Jackson Laboratories, Bar Harbor, ME (strain name = *B6*.*129P2-Il10*^*tm1Cgn*^*/J*; stock # 002251 and strain name = *B6*.*129S6-Tnf*^*tm1Gkl*^*/J*; stock # 005540). The same parent lines were crossed with *Aicda*^-/-^ mice that had been extensively back-crossed onto the C57BL/6 background (obtained from their creator, Dr. Tasuku Honjo; [21]) to create mice that were homozygous for TNF, IL-10, and AID deficiency (*Tnf*^-/-^, *Il10*^-/-^, *Aicda*^-/-^; termed “TIA” mice). Since mice deficient in TNF and IL10 develop UC-like disease at or soon after weaning and reproduce poorly [22], these lines were typically maintained by breeding T/I, *Tnf*^-/-^ *Il10*^+/-^ (T/I^het^), or *Tnf*^-/-^ *Il10*^+/-^ *Aicda*^-/-^ (TI^het^A) males to T/I^het^ or TI^het^A females. We showed previously that T/I^het^ mice did not develop colitis [22]; therefore T/I^het^ mice produced as littermates to and co-housed with T/I mice were used for biomarker studies as non-colitis controls.

Mice were housed in polycarbonate micro-isolator cages in individually ventilated racks under barrier conditions that excluded helicobacter and norovirus as well as other known mouse pathogens. Mice had *ad libitum* access to PicoLab Mouse Diet 20/5058 (LabDiet, St. Louis, MO, USA) and water. Sentinel mice exposed repetitively to dirty bedding from the mice used in this study were negative for parasites by microscopic examination, negative for *Citrobacter rodentium* by fecal culture, negative for infection with *Helicobacter* species by PCR of feces and negative by serology for a panel of 22 murine protozoal, bacterial and viral pathogens, including murine parvovirus, murine hepatitis virus, and murine norovirus (S1 Table).

Mice were euthanized by CO2 asphyxiation for assessment of colitis severity and neoplasia when they either reached the pre-defined study endpoint of 28 weeks of age or met humane endpoints of >15% loss of body weight, rectal prolapse, or other clinical signs of pain and distress, including hunching, shivering, minimal movement on gentle prodding, or grimace [23]. Once respiration ceased, death was assured by exsanguination, harvest of vital organs, and/or decapitation. Although colitis has the potential to produce pain and distress, analgesics were not employed in these studies, since non-steroidal anti-inflammatory agents (NSAIDs) can exacerbate colitis and opioids affect intestinal motility and function. Monitoring, including determination of body weight, generally occurred daily, but was always provided at least 3 times weekly. Suffering was minimized and unattended deaths were prevented by providing euthanasia as soon as possible (≤ 2 hours) for mice that met humane endpoints. All mice involved in this study were euthanized, either at defined time points or when they met humane endpoints, which defined the mortality described in this study. There were no unattended natural deaths.

### Tissue analysis

After euthanasia, the entire digestive tract was dissected out from stomach to anus. A segment of mid-jejunum and the entire colon were routinely submitted for histologic examination for all mice. The lower stomach/duodenum and mesenteric lymph nodes from TIA mice were also routinely sampled for histologic examination. Five colors of permanent tissue marking dye (Bradley Products, Bloomington, MN) were used to identify specific colonic regions (cecum, proximal, mid-, and distal colon, and terminal colon/rectum). Intact, unopened intestinal segments were fixed in Carnoy’s solution for 2–4 hrs then embedded in paraffin. This method is ideal for identification of flat neoplastic lesions, since it allows examination of 2 sides of the colon and the presence of feces prevents tissue curling during fixation but does not interfere with most slide-based assays. Hematoxylin and eosin (H&E)-stained sections were evaluated by a board-certified pathologist who was blinded to tissue identity/genotype. The severity of inflammation was scored as previously described [15], using a scale from 0–75 that takes into account mucosal changes such as hyperplasia and ulceration, degree of inflammation, and percentage of each bowel segment affected by these changes. Using this scale, a score 0–12 indicates the absence of colitis, 13–24 indicates mild, and ≥ 25 indicates moderate to severe colitis. Animals with histologic scores that fall into the moderate to severe range typically have either scattered severe inflammatory lesions or extensive disease involving the mucosa. Sections were also scored for non-invasive or invasive neoplasia [24]. Gastrointestinal intraepithelial neoplasia (synonymous with atypical hyperplasia, microadenoma, carcinoma in situ) and adenoma were considered to be non-invasive lesions. A diagnosis of invasive carcinoma required the presence of a desmoplastic response (formation of an abundant collagenous stroma) to differentiate invasion from mucosal herniation or pseudoinvasion. Regions of neoplasia that were separated by regions of normal mucosa were scored as separate lesions.

### Serum biomarker levels

Serum was obtained from eight to 10 week old T/I, TIA, and T/I^het^ mice immediately following euthanasia. Serum biomarker profiling (cytokines, chemokines, immunoglobulins) was performed in the Duke Regional Biocontainment Laboratory (RBL) Immunology Unit (Durham, NC) under the direction of Dr. Gregory D. Sempowski, using Luminex bead-based multiplex immunoassays (BioRad), according to the manufacturer’s instructions.

### Statistical analysis

Statistical comparison of histologic scores was performed using the Mann Whitney U test. Serum cytokine/chemokine measurements from T/I, TIA, and control T/I^het^ mice were log-transformed and compared via 2-way ANOVA with Tukey’s test for multiple comparisons (GraphPad Prism, version 8.4.3). Categorical data was compared via Fisher’s exact test. Survival rates were determined by Kaplan-Meier analysis, with p-values calculated using the log rank test (MediCalc Software, Version 13.3, Mariakerke, Belgium). A Poisson regression model was used to determine the effect of genotype on number of neoplastic lesions, while adjusting for age (R statistical program). A p value ≤ 0.05 was considered to represent a significant difference between groups.

### Ethics statement

All animal studies were approved (protocol numbers A151-09-05, A093-12-04, or A43-15-02) by the Institutional Animal Care and Use Committee of Duke University, an institution accredited by the Association for Assessment and Accreditation of Laboratory Animal Care (AAALAC), International.

## Results

### Spontaneous colon inflammation is rare in *Aicda*-deficient mice

Mice that were deficient in *Aicda alone* demonstrated no grossly discernable phenotype over the course of this study. 100% of a cohort of 16 *Aicda*^-/-^ mice survived to the experimental endpoint of 28 weeks. Most of the mice in the cohort had no colon inflammation (S1 Fig). However, one mouse in this group demonstrated focal mild to moderate mucosal hyperplasia and inflammation that scored as mild colitis (histologic score = 16). This led to a mean ± SD histologic score = 3 ± 4 for this genotype (n = 16).

### *Aicda* deficiency does not prevent the development of UC-like colitis in TIA mice

**Fig 1:**
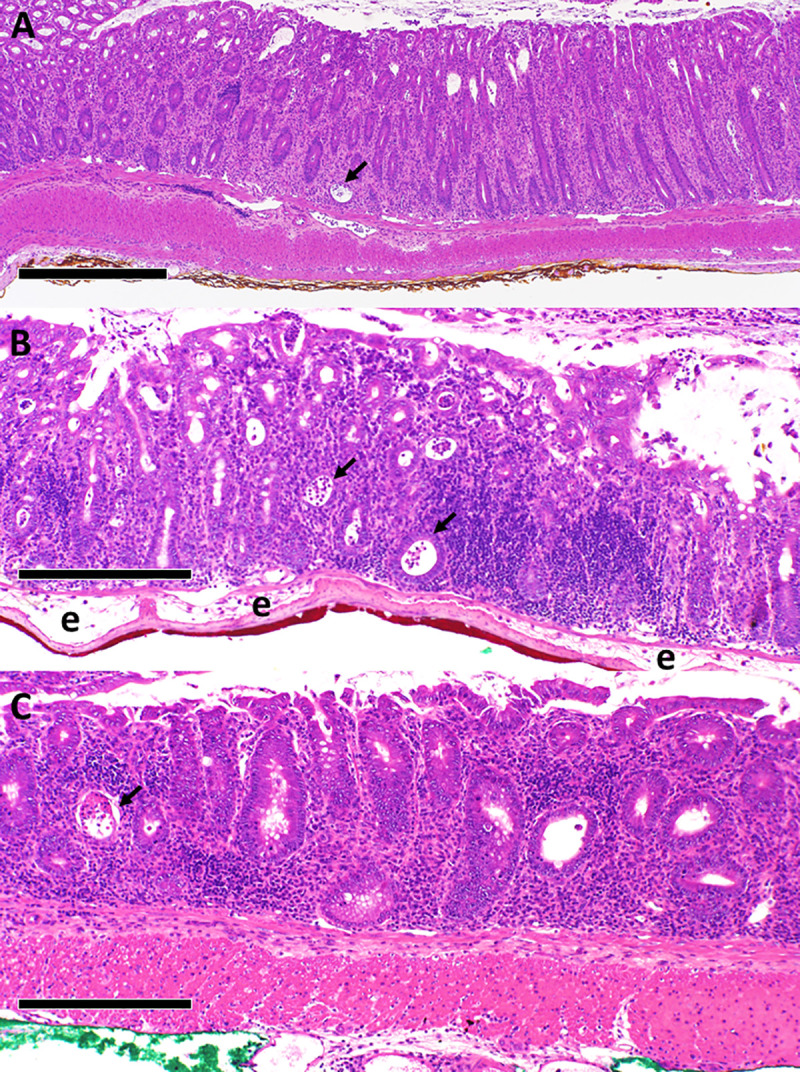
Histology of colitis in TIA mice. TIA mice generally presented with moderate to severe pan-colitis, primarily involving the mucosa, as shown in these representative fields derived from the terminal colon/rectum (**A**), distal colon (**B**), and mid-colon (**C**). The lamina propria is packed with inflammatory cells. Representative crypt abscesses are indicated by arrows. “e” in panel **B** denotes areas of edema in the submucosa. Scale bar represents 500 μm in **A** and 250 μm in **B** and **C**.

**Fig 2:**
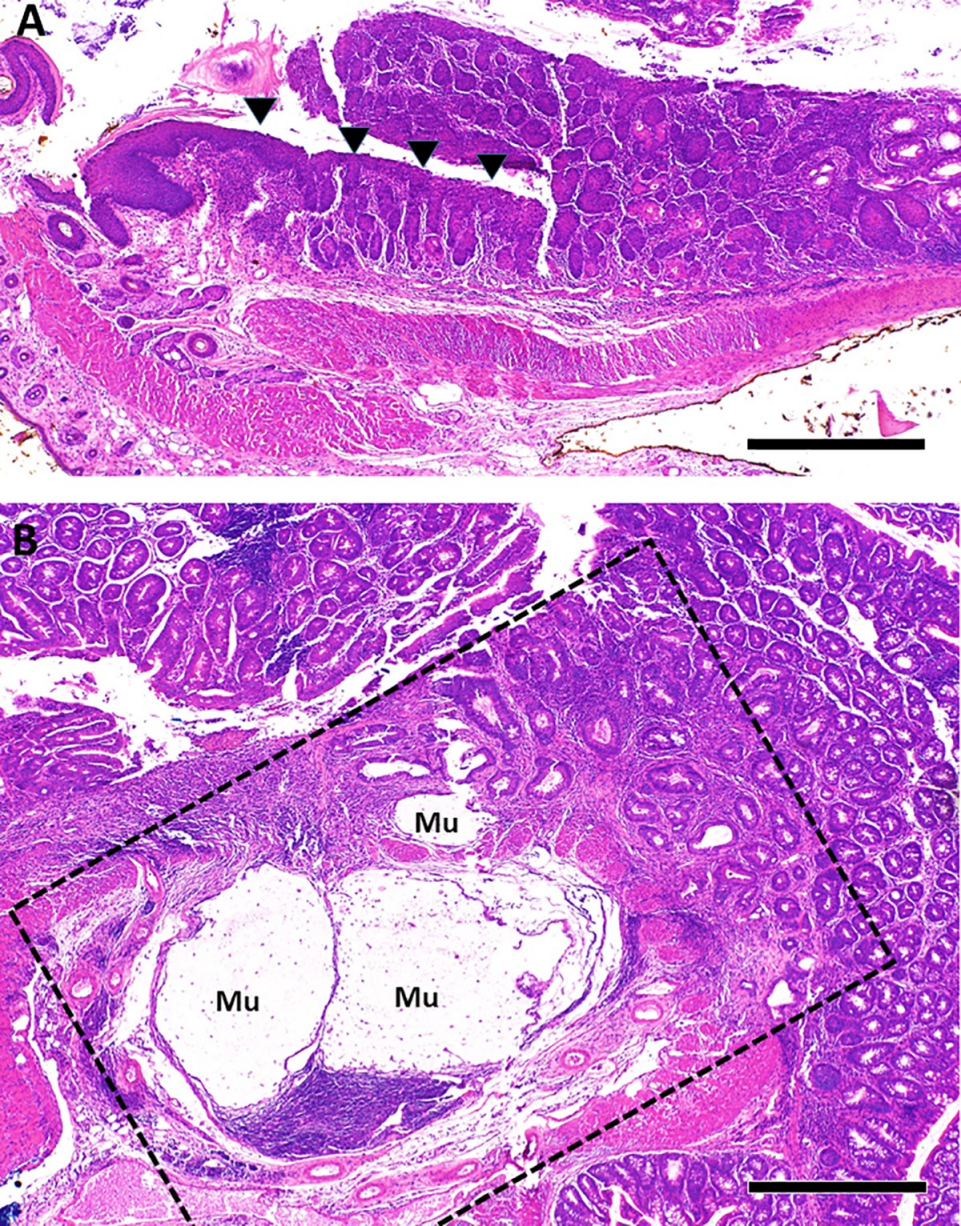
Squamous metaplasia and neoplasia in TIA mice. **A**. Squamous metaplasia (arrowheads) was common in the rectum of TIA mice, shown here in a 9 week old. **B**. The dotted lines highlight a focus of invasive mucinous adenocarcinoma identified in the proximal colon of a 15 week old TIA mouse with severe pan-colitis (histologic score = 55). “Mu” indicates mucin lakes associated with invasive carcinoma. Scale bar in **A** and **B** represents 500 μm.

We previously reported that T/I mice spontaneously developed moderate to severe UC-like colitis soon after weaning [20]. All T/I mice that were additionally *Aidca*-deficient (TIA mice) also spontaneously developed moderate to severe UC-like colitis (mean ± SD histologic score = 49 ± 9; n = 77). As in human UC, inflammation involved the entire colon (“pan-colitis”), including the cecum (S2 Fig). Severe colitis was uniformly present in all 6–7 week old TIA mice examined (mean ± SD histologic score = 53 ± 6; n = 11), consistent with onset soon after weaning. UC-like colitis in TIA mice (Fig 1) was qualitatively indistinguishable from that seen in T/I mice (S3 Fig and [20]). For both genotypes, the lamina propria of all inflamed regions was packed with inflammatory cells with frequent crypt abscesses and ulceration and inflammation generally was confined to the mucosal layer and did not extend deep into the muscle wall (Fig 1). Squamous metaplasia of the rectum was common, appearing in 36 of 69 (52%) evaluable TIA mice (Fig 2A).

To begin to identify potential biomarkers that could distinguish colitis in T/I versus TIA mice, concentrations of a panel of 28 serum cytokines and chemokines were measured in serum from 8–10 week old T/I (n = 28) and TIA mice (n = 17) (Table 1). Eight to 10 week old T/I^het^ mice (n = 13) were also studied as non-colitis controls. Results showed that TIA mice had increased serum IL1α and decreased IL12p40 and M-CSF compared with T/I mice. Levels of 25 other cytokines and chemokines tested (Table 1) were similar in T/I and TIA mice. Serum levels of GM-CSF, IFN-γ, IL6, IL17, IP10, MCP-1, MIP1α, and MIG were higher and serum levels of IL1α, IL5, IL12(p70), MCP1, and MIP1β were lower in T/I mice compared to their non-inflamed T/I^het^ littermates (Table 1). Similar differences were seen between TIA and control T/I^het^ mice for most of these biomarkers, with the exception that serum levels of IL12(p70) and MCP-1 in TIA mice remained similar to control (Table 1).

**Table 1:** Serum cytokine and chemokine levels in T/I, TIA, and control T/I^het^ mice^a^.

	T/I mice (n = 28)	TIA mice (n = 17)	Control T/I^het^ mice (n = 13)
Eotaxin	918 (471–1607)	928 (559–1388)	954 (412–1760)
GM-CSF	16 (8–30)	16 (8–37)	4 (2–38)^b^^,^^c^
IFN-γ	38 (4–524)	43 (2–236)	4 (2–11) ^b^^,^^c^
IL1α	112 (28–1883)	157 (23–1683)*	192 (5–1605) ^b^^,^^c^
IL1β	2 (2–12)	2 (2–265)*	4 (1–10)
IL2	5 (2–8)	2 (2–9)	6 (2–11)
IL3	2 (2–4)	2 (2–8)	2 (2–22)
IL4	2 (2–11)	2 (2–42)	2 (2–2)
IL5	2 (2–11)	2 (2–79)	12 (2–30) ^b^^,^^c^
IL6	19 (2–44)	36 (4–104)	2 (2–8) ^b^^,^^c^
IL7	2 (2–84)	2 (2–26)	2 (2–14)
IL9	47 (10–487)	69 (10–245)	46 (46–106)
IL12p40	12 (2–98)	7 (2–14)*	9 (2–30)
IL12p70	4 (1–156)	8 (1–211)	8 (7–45) ^b^
IL13	154 (9–350)	186 (69–913)	91 (58–206)
IL15	29 (8–1378)	27 (8–1909)	54 (22–135)
IL17	26 (4–159)	60 (7–146)	2 (2–7) ^b^^,^^c^
LIF	2 (2–16)	2 (2–17)	2 (2–2)
LIX	4634 (2397–7787)	3900 (809–7336)	5930 (544–7824)
IP10	292 (120–670)	355 (150–916)	140 (27–334) ^b^^,^^c^
KC	175 (44–765)	189 (16–1226)	122 (68–211)
MCP1	29 (9–58)	27 (9–479)	46 (24–60) ^b^
MIP1α	31 (10–62)	29 (10–258)	24 (1–33) ^b^
MIP1β	9 (9–28)	9 (9–22)	36 (9–45) ^b^^,^^c^
M-CSF	20 (7–517)	11 (6–44)*	12 (6–33)
MIP2	80 (20–606)	77 (37–126)	87 (32–132)
MIG	302 (68–2448)	368 (51–907)	49 (30–138) ^b^^,^^c^
RANTES	36 (8–328)	21 (6–84)*	29 (16–65)

^a^ Values shown represent the median (range) of cytokine/chemokine in pg/ml in cohorts of 8–10 week old mice.

^b^ Indicates p < 0.05 for comparison of control T/I^het^ versus T/I mice.

^c^ Indicates p < 0.05 for comparison of control T/I^het^ versus TIA mice.

* Indicates p < 0.05 for comparison of T/I versus TIA mice.

**Fig 3:**
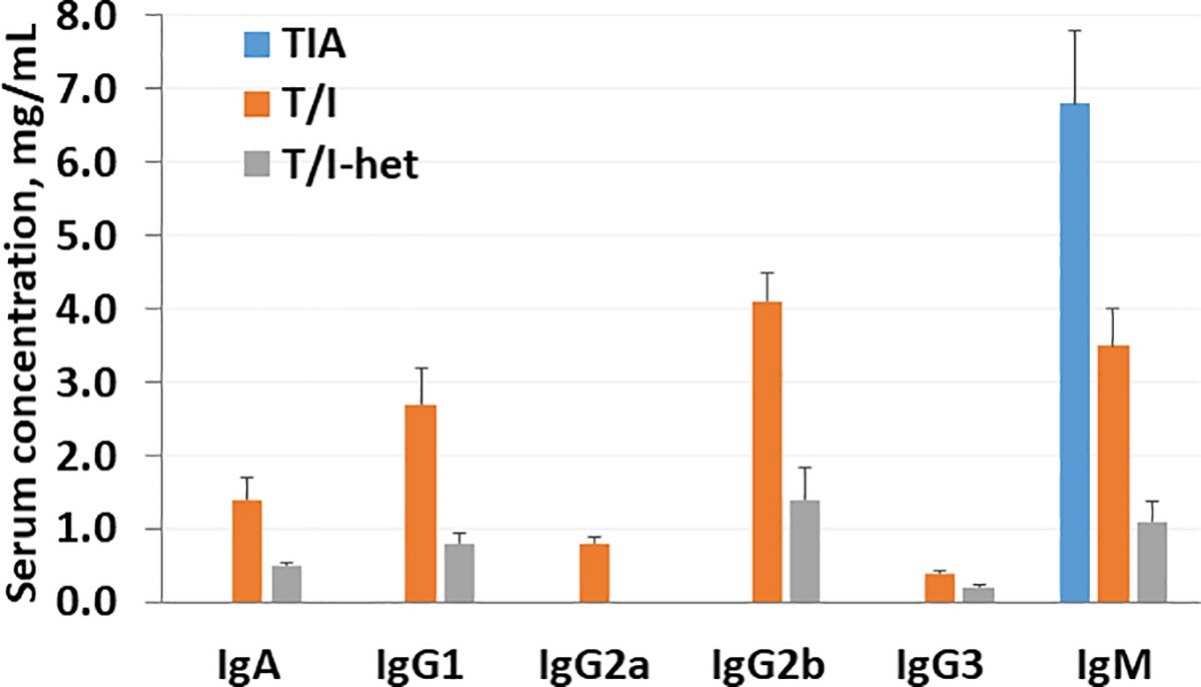
Serum immunoglobulin concentrations in TIA, T/I, and control T/I^het^ mice. The serum concentration of various immunoglobulin isotypes are shown for 8–10 week old TIA (n = 17), T/I (n = 28), and control T/I^het^ mice (n = 13). Values shown represent the mean ± standard error of the mean (SEM). Values obtained for TIA mice were statistically different than for T/I mice for all isotypes tested, with p values ranging from p = 0.006 for IgM to 7 x 10^−12^ for IgG3 (Student’s t-test*)*.

To further assess immune system function, serum immunoglobulins were measured in this same cohort. Total serum immunoglobulin (Ig) was decreased in TIA mice (mean ± SEM = 6.8 ± 1.0 mg/ml) compared with T/I mice (12.1 ± 1.1 mg/ml; p = 0.0005). However, IgM levels were increased in TIA mice relative to T/I mice (p = 0.006) and TIA mice had undetectable levels of IgA, IgG1, IgG2a, IgG2b, and IgG3 (Fig 3). This phenotypic pattern of Ig expression results from a deficiency of Ig class switching and is diagnostic of hyper-IgM syndrome [25]. Levels of all immunoglobulin isotypes were increased in T/I mice relative to their non-inflamed T/I^het^ littermates (p values from 0.002 for IgG1 to 1.3 x 10^−7^ for IgG2a; Fig 3).

### *Aicda* deficiency increases mortality in T/I mice with UC-like colitis

**Fig 4:**
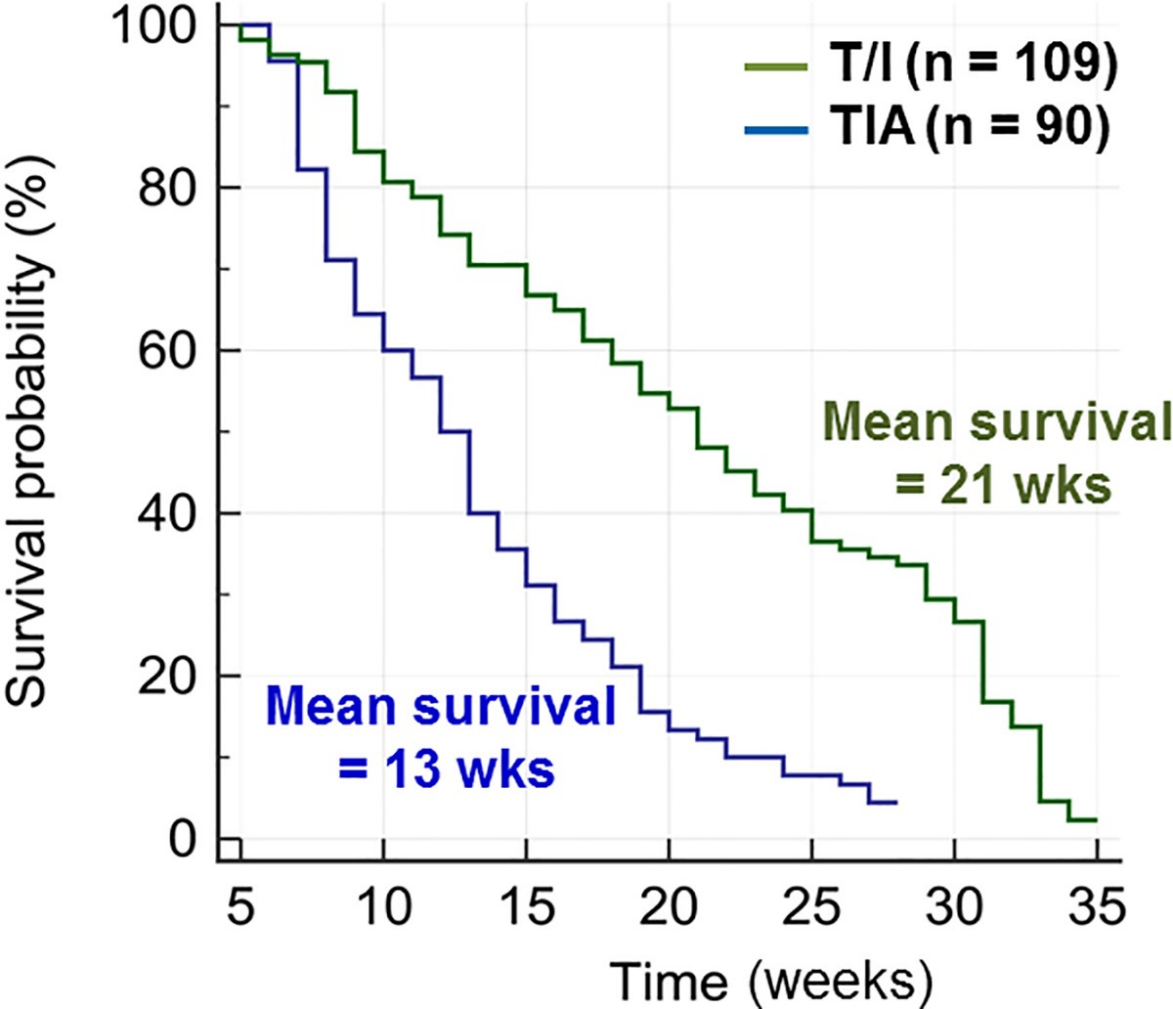
Survival of T/I and TIA mice as a function of age. To prevent unnecessary suffering, morbidity that triggered humane endpoints was used as a surrogate for mortality, as described in Materials and Methods. Kaplan-Meier analysis demonstrates that TIA mice have a significantly shorter mean survival (13 weeks) than T/I mice (21 weeks; p = 0.0001, log-rank test).

We next assessed the effect of *Aicda* deficiency on mortality by comparing survival of T/I versus TIA mice. As shown in Fig 4, the mean survival of TIA mice was 13 weeks (median 12 wks; 95% CI, 10–14 weeks; n = 90), while the mean survival of T/I mice was 21 weeks (median 21 wks; 95% CI, 18–24 weeks; n = 109) (p < 0.0001, log rank test). Excess mortality of TIA mice was observed beginning at 8 weeks of age and typically manifested as weight loss or other markers of pain or distress rather than as rectal prolapse.

### Effect of *Aicda* deficiency on colorectal neoplasia in mice with UC-like colitis

**Fig 5:**
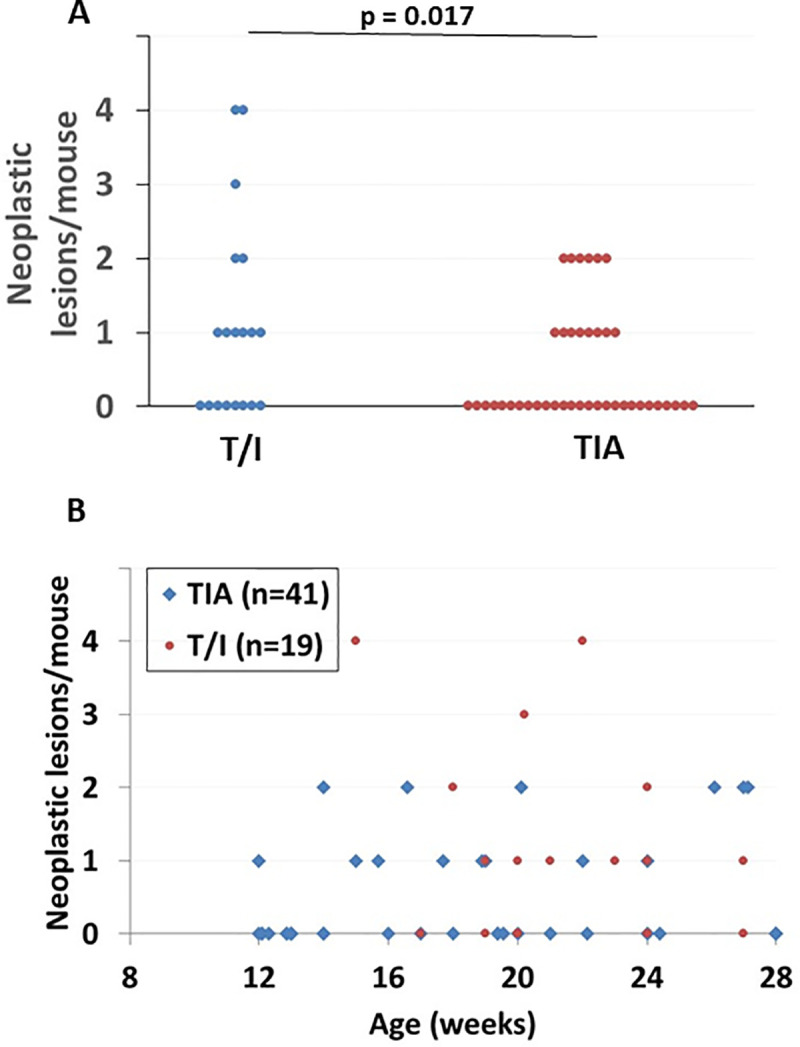
Incidence and multiplicity of colonic neoplasia in T/I versus TIA mice. **A.** The number of tumors per mouse is shown for T/I (n = 19) and TIA mice (n = 41). Each point represents a single mouse. The p value shown is the result of a multivariable analysis that adjusted for age when neoplasia was ascertained (Poisson regression analysis). **B.** The number of tumors per mouse is shown as a function of age at euthanasia for the same cohort illustrated in panel **A**. Each point represents a single mouse, however some points are not visible due to multiple mice with the same age and tumor count.

To assess the effect of *Aicda* deficiency on the incidence of colorectal neoplasia, cohorts of T/I (n = 19) and TIA mice (n = 41) were closely monitored between ages of 12 and 28 weeks and euthanized at age 28 weeks or when they met humane endpoints, whichever came first. Of the 19 T/I mice studied, eleven (58%) had colorectal neoplasia, with a mean of 2 neoplastic lesions/mouse with neoplasia (range = 1–4) (Fig 5). Of the 41 TIA mice studied, fourteen (34%) exhibited colorectal neoplasia (p = 0.10 vs. T/I, Fisher’s exact test), with a mean of 1 neoplastic lesion/mouse with neoplasia (range = 1–2; p = 0.06 vs. T/I) (Fig 5). However, since the TIA cohort was younger than the T/I cohort at the time of study (TIA = 19 ± 5 weeks vs T/I = 21 ± 3 weeks; p = 0.03), it was important to rule out that the trends toward decreased incidence and multiplicity of neoplasia in TIA mice were due to insufficient time for neoplasia to become evident before euthanasia was required. Multivariable analysis that adjusted for age when neoplasia was ascertained showed a significant decrease in the numbers of neoplastic lesions in TIA compared with T/I mice (p = 0.017; Poisson regression analysis).

Neoplastic lesions in TIA mice ranged from gastrointestinal intraepithelial neoplasia to adenocarcinoma that invaded through the serosa (Fig 2B). No evidence of metastasis was found grossly in the liver or in any of the lymph nodes examined histologically. All TIA mice in this cohort demonstrated moderate to severe colitis (S4 Fig), with a trend toward higher histologic scores (mean ± SD = 48 ± 10; n = 41), compared with 41 ± 13 (n = 19) in the T/I group (p = 0.06).

### Other histologic findings in TIA mice

**Fig 6:**
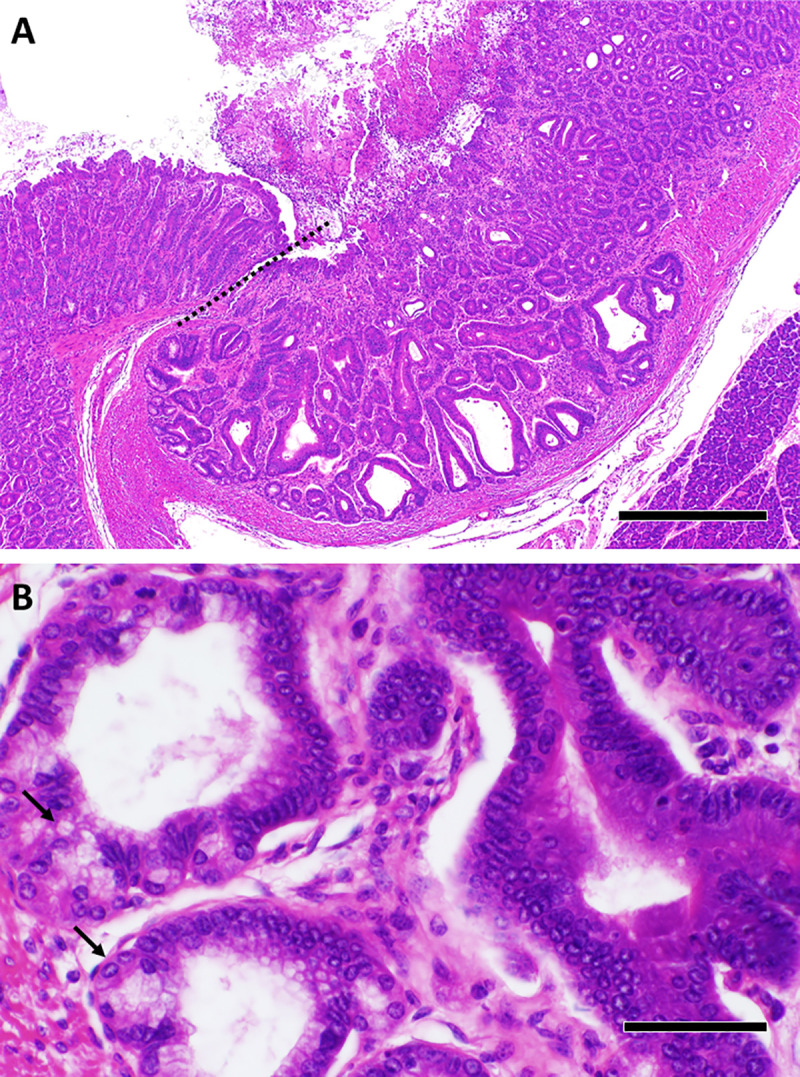
Colonic metaplasia of Brunner’s glands and duodenal inflammation in TIA mice. **A**. The pyloric region of the glandular stomach (left of dashed line) and the duodenum (right of dashed line) are shown. The duodenal mucosa demonstrates mucosal epithelium and Brunner’s glands with loss of villous architecture and histology similar to that seen in the colon (colonic metaplasia). **B**. A higher magnification views demonstrates the metaplastic epithelium and surrounding lamina propria and submucosa with marked chronic and active inflammation, similar to what was observed in the colon of these mice. Images shown are from a 9 week mouse, where some residual cells (arrows) exhibit morphology typical of Brunner’s glands. Older mice with such metaplasia typically lacked residual identifiable Brunner’s glands. Scale bar represents 500 μm in **A** and 50 μm in **B**.

**Fig 7:**
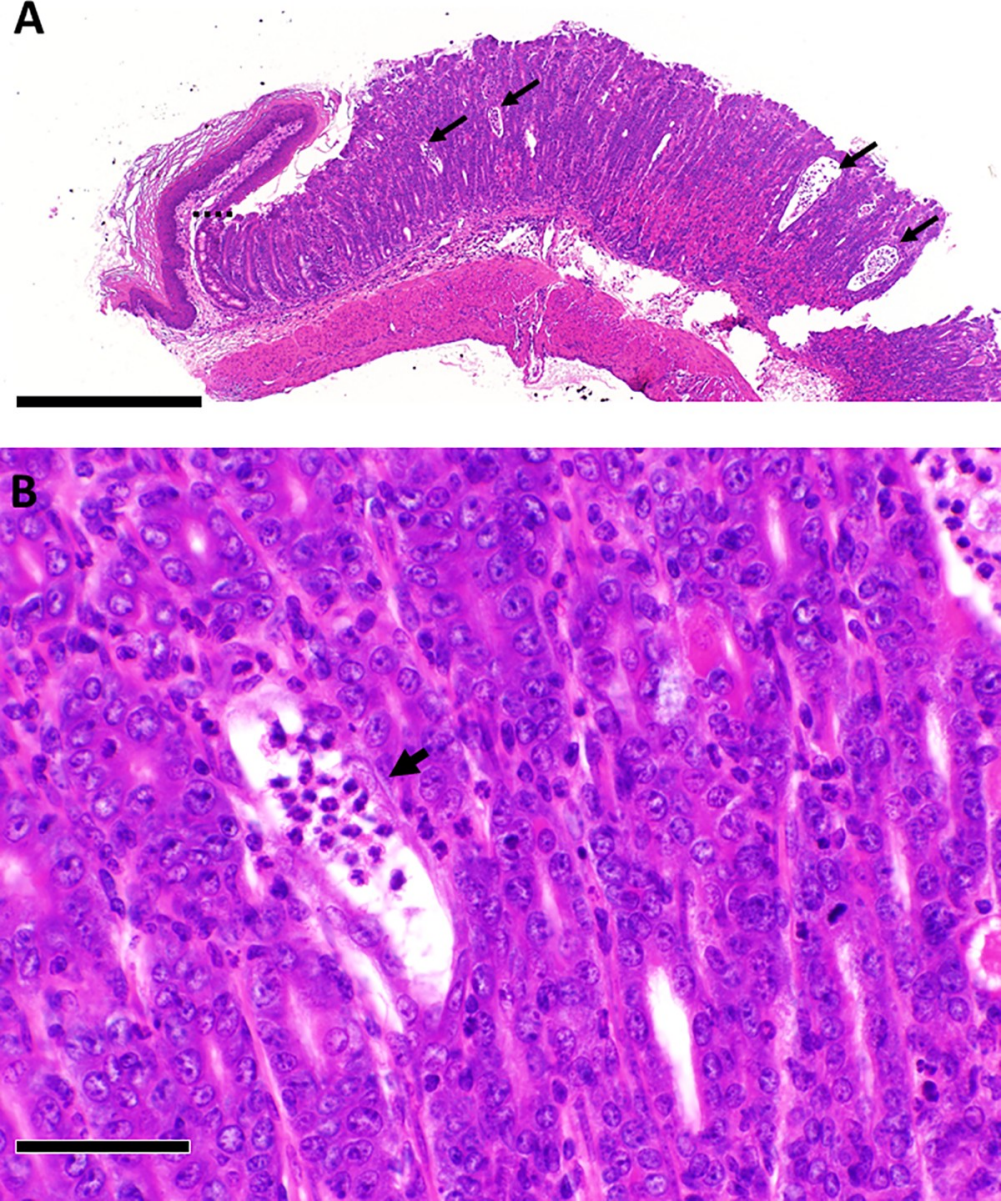
Stomach inflammation in TIA mice. The dashed line in **A** represents the boundary between the forestomach and the glandular stomach. A higher magnification view in B shows inflamed gastric glands that resemble crypt abscesses, with thinning of glandular epithelium and neutrophils present within the dilated lumens, as indicated by arrows. Scale bar represents 500 μm in **A** and 50 μm in **B**.

In addition to colitis with or without colonic neoplasia as described above, TIA mice frequently exhibited grossly evident swelling at the gastroduodenal junction. Histologic examination of this area revealed a change in the epithelium of the duodenal surface mucosa and submucosal Brunner’s glands to resemble colonic epithelial cells (“colonic metaplasia”; Fig 6) in 42 of 66 evaluable TIA mice (64%). This metaplastic change was typically associated with acute and chronic mucosal inflammation that was limited to the short segment of duodenum. Random sampling showed no evidence of inflammation in the mid-jejunum of these animals. Inflammation was also occasionally seen in the gastric glands and in the lamina propria of the glandular stomach in a subset of TIA mice, with a histology that resembled the crypt abscesses seen in the colon (Fig 7). Mesenteric lymph nodes were often massively enlarged, consistent with the severe colon inflammation observed, but the protruding lymphoid follicular structures previously described to be present on the antemesenteric side of duodenal and jejunal segments in singly *Aicda*-deficient mice [26] were not typically seen along the small intestine of TIA mice.

## Discussion

The studies reported here show that TIA mice that are triply deficient in *Il10*, *Tnf*, and the APOBEC family mutator enzyme *Aicda* develop severe UC-like colitis soon after weaning, similar to T/I mice that are deficient in *Tnf* and *Il10* alone. Multivariable analysis that adjusted for age when neoplasia was ascertained showed that deletion of the *Aicda* gene decreased the development of inflammation-associated colorectal neoplasia, with decreased numbers of neoplastic colorectal lesions and a trend toward decreased incidence of neoplasia in TIA mice. Inflammation severity was statistically similar in T/I and TIA mice, with a trend toward slightly increased histologic scores in TIA mice. Serum biomarker analysis revealed that TIA mice had defective Ig class-switching that resulted in hyper-IgM syndrome, as well as increased serum IL1α and slightly decreased IL12(p40) and M-CSF compared with T/I mice. Taken together, these studies show that *Aicda* deficiency can decrease but is not sufficient to prevent inflammation-associated colon neoplasia in the setting of severe UC-like inflammation. The generally similar cytokine profiles in TIA and T/I mice, combined with similar histologic severity and extent of inflammation in the colon, strongly suggest that the decreased colorectal neoplasia observed in TIA relative to T/I mice is due to direct or indirect effects of *Aicda* deficiency that affect mechanisms of carcinogenesis, rather than simply affecting inflammation severity.

Previous research showed that the expression of the mutation-inducing enzyme encoded by *Aicda* is induced by colon inflammation and can enhance the acquisition of p53 mutations by colon epithelial cells [6, 19]. This is important since, in contrast to what is observed in sporadic colon cancers, p53 mutations occur early in UC, with higher frequencies in inflamed vs. non-inflamed regions of the colon [27, 28]. Consistent with the lack of early *WNT*-*APC* mutations that stimulate polypoid growth (reviewed in [27, 29]), the colorectal cancers that occur in IBD patients typically develop from non-polypoid (“flat”) mucosa and progress rapidly. Takai *et al*. previously showed that although *Aicda* deficiency did not affect inflammation severity based on cytokine production in *Il10*^-/-^ mice, it decreased the frequency of nucleotide alterations in the *Trp53* gene [19]. However, *Aicda* deficiency was not sufficient to prevent oncogenic mutagenesis in the setting of ongoing inflammation in their model, since although it decreased the percentage of *Il10*^-/-^ mice that developed invasive colon cancers, it did not affect the percentage of *Il10*^-/-^ mice with dysplastic precursor lesions such as adenomas at one year of age [19]. Similarly, we found that the severe inflammation present throughout the colon in our UC-like model continued to drive the development of neoplasia by 28 weeks of age or earlier, even in the setting of *Aicda* deficiency. The more limited anti-neoplastic effects of *Aicda* deficiency observed in our study compared with Takai *et al* [19] may reflect the increased proportion of colon epithelial cells that were exposed to inflammation-associated mutagenesis in our geographically continuous UC-like colitis models compared to the focal CD-like colon inflammation typically characteristic of mice deficient in IL10 alone. Other murine models of carcinogenesis, such as the azoxymethane plus dextran sulfate sodium model, may be useful for further elucidating mechanisms by which *Aicda* deficiency may decrease inflammation-associated colon carcinogenesis.

The absence of *Aicda* in all cells of the TIA mice we studied led to hyper-IgM syndrome, similar to when humans have bi-allelic lack-of-function mutations in *AICDA* [25]. Hyper-IgM syndrome is characterized by defective immunoglobulin class-switch recombination as well as defective somatic hypermutation of B cell antigen receptors. Interestingly, although the single knockout *Aicda*^-/-^ mice housed in our specific pathogen-free facility showed no evidence of colitis, IBD has been reported in humans with *AICDA* mutations [30]. *Aicda*^-/-^ and *Aicda*-mutant mice were previously shown to develop grossly evident hyperplasia of small intestinal lymphoid follicles, along with expansion of microbiota in the small intestine [26, 31]. However, in our study, mice singly deficient in *Aicda* showed no evidence of small intestinal lymphoid follicular hyperplasia and minimal to no colitis. This lack of phenotype may be due to the relative cleanliness of our specific pathogen-free environment (S1 Table), which we have also shown to be sufficient to prevent the development of colitis in mice deficient in *Il10*^-/-^ alone, in the absence of specific triggering [13, 32]. TIA mice demonstrated enlarged mesenteric lymph nodes consistent with their severe colitis, but did not exhibit the small intestinal follicular hyperplasia reported by Fagarson *et al* [26]. Instead, many TIA mice exhibited colonic metaplasia in the duodenum that often developed inflammation similar to what was seen in the colon of these mice. The mechanisms underlying this pathology will require additional study, but may include local changes in abundance and type of microbiota, changes in gut permeability related to lack of IgG or IgA, the lack of somatic hypermutation to modify antibody repertoire to shape the relative composition and distribution of microbiota, systemic invasion of gut microbes, and/or other mechanisms. However, the generally similar severity of colitis in T/I and TIA mice, as evidenced by similar histologic scores of inflammation severity and similar levels of key pro-inflammatory cytokines and chemokines definitively shows that the abilities to carry out somatic hypermutation and immunoglobulin class-switching to produce IgG and IgA do not protect against the development of colitis in the setting of TNF and IL10 deficiency.

Although most aspects of inflammation seen in T/I and TIA mice very closely resemble those seen in human UC, the colonic metaplasia of Brunner’s glands and the duodenal inflammation we observed have not previously been reported in humans with UC. Additional studies will be required to fully assess the importance of these observations and the responsible mechanisms. The squamous metaplasia that was frequently observed in the rectum of TIA mice may be related to their lack of TNF, since squamous metaplasia of the rectum has also been observed in other strains of mice that are genetically TNF-deficient [20] as well as in wild type mice that were treated with an inhibitor of TNF transcription [33].

Since the experiments reported here used mice globally deficient in *Aicda*, it is not possible to determine whether the phenotypic differences between T/I and TIA mice are directly caused by AID deficiency in the gut epithelium, are a consequence of inefficient humoral adaptive immune system due to the effects of AID deficiency on B cells that severely alter immunoglubulin levels and/or repertoires in both the Peyer's patches and the lumen of the gut, and/or are due to other mechanisms. Follow-up studies using mice with targeted tissue-specific knockout of *Aicda* will be required to address these issues. Determining which cells must be AID-deficient to decrease the frequency of inflammation-associated colorectal neoplasia has important clinical implications, since this may allow the development of therapeutic interventions that decrease the incidence of colorectal neoplasia without causing undesirable changes in serum antibodies and gut microbiota.

In summary, the studies reported here show that *Aicda* deficiency can decrease, but is not sufficient to prevent, the risk of inflammation-associated colorectal neoplasia in the setting of severe UC-like inflammation. The TIA model may be useful for assessing the roles of antibody class-switch recombination deficiency and somatic hypermutation on regulation of microbiota in the small intestine and colon, as well as the pathogenesis of colitis associated with hyper-IgM syndrome in humans. Further studies will be required to determine the mechanisms that drive early mortality in TIA mice.

## Supporting information

S1 Fig Colon histology typical of mice deficient in *Aicda* alone. Fields shown are from the terminal colon/rectum (**A**), distal colon (**B**), and mid-colon (**C**). Scale bar represents 100 μm.
(TIF)

S2 Fig Histologic scoring of colitis in TIA mice. The severity of inflammation was scored as previously described [15], using a scale that takes into account mucosal architectural changes (M), degree of inflammation (I), and percentage of each bowel segment affected by any and severe changes (E1, E2). The bars indicate the mean ± SD score for the 5 colon segments examined in each mouse: terminal colon/rectum, distal colon, mid-colon, proximal colon, and cecum. The scores for each segment are summed to provide the overall histologic score of 49 ± 9 observed for TIA mice (n = 77), as described in Results. For the M score, 0 = no significant lesions, 1 = mild epithelial hyperplasia, 2 = moderate epithelial hyperplasia, and 3 = severe epithelial hyperplasia, with crypt branching or herniation. For the I score, 0 = no inflammation, 1 = mild inflammation limited to the mucosa, 2 = moderate inflammation present in mucosa and submucosa, 3 = severe inflammation with obliteration of normal architecture, erosions, and/or crypt abscesses, and 4 = level 3 changes plus ulceration. The E1 score is derived from the percent of the segment affected in any manner. The E2 score is derived the percent of the segment with level 3 or 4 changes. For the E1 and E2 scores, 1 = <5% of segment affected, 2 = 5–30% of segment affected, 3 = 31–60% of segment affected, and 4 = >60% of segment affected. Since the total histologic score is derived from summing (M + I + E1 +E2) scores from the 5 segments examined, the maximum score is 75.
(TIF)

S3 Fig Histology of colitis in T/I mice. T/I mice generally developed moderate to severe mucosal inflammation involving cecum to rectum (“pan-colitis”) soon after weaning, as described in [20]. The representative fields shown are from the terminal colon/rectum (**A**), distal colon (**B**), and mid-colon (**C**). Marked epithelial hyperplasia is present and the lamina propria is packed with inflammatory cells. Representative crypt abscesses are indicated by arrows. Scale bar represents 250 μm in panels A and B and 500 μm in panel C.
(TIF)

S4 Fig Histologic scores in T/I versus TIA mice. Graph shows histologic scores for T/I (n = 19) and TIA mice (n = 41) between the ages of 12 and 28 weeks who were euthanized for determination of neoplasia, either when they met humane endpoints or at the experimental endpoint of 28 weeks. Each point represents a single mouse. p = 0.06 (Student’s t-test).
(TIF)

S1 Table Pathogen status of mice used for these studies. (DOCX)
