# Supplementary figures and images for: Deficiency of activation-induced cytidine deaminase in a murine model of ulcerative colitis
Source: PLoS One. 2020 Sep 17;15(9):e0239295. doi: 10.1371/journal.pone.0239295 (PMC7498091; doi:10.1371/journal.pone.0239295)

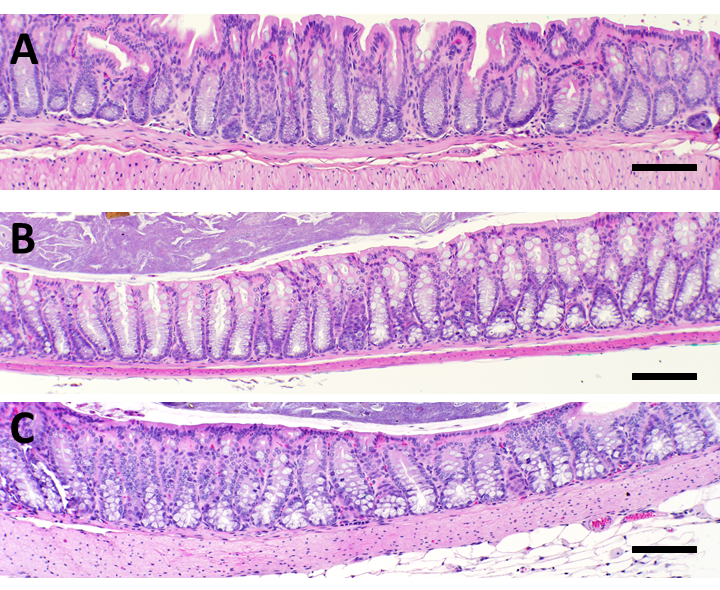

Supplement: S1 Fig — Fields shown are from the terminal colon/rectum (A), distal colon (B), and mid-colon (C). Scale bar represents 100 μm. (TIF) [file pone.0239295.s001.tif]

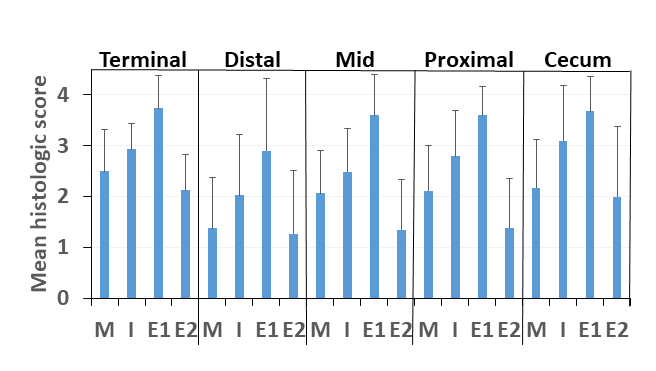

Supplement: S2 Fig — The severity of inflammation was scored as previously described [15], using a scale that takes into account mucosal architectural changes (M), degree of inflammation (I), and percentage of each bowel segment affected by any and severe changes (E1, E2). The bars indicate the mean ± SD score for the 5 colon segments examined in each mouse: terminal colon/rectum, distal colon, mid-colon, proximal colon, and cecum. The scores for each segment are summed to provide the overall histologic score of 49 ± 9 observed for TIA mice (n = 77), as described in Results. For the M score, 0 = no significant lesions, 1 = mild epithelial hyperplasia, 2 = moderate epithelial hyperplasia, and 3 = severe epithelial hyperplasia, with crypt branching or herniation. For the I score, 0 = no inflammation, 1 = mild inflammation limited to the mucosa, 2 = moderate inflammation present in mucosa and submucosa, 3 = severe inflammation with obliteration of normal architecture, erosions, and/or crypt abscesses, and 4 = level 3 changes plus ulceration. The E1 score is derived from the percent of the segment affected in any manner. The E2 score is derived the percent of the segment with level 3 or 4 changes. For the E1 and E2 scores, 1 = <5% of segment affected, 2 = 5–30% of segment affected, 3 = 31–60% of segment affected, and 4 = >60% of segment affected. Since the total histologic score is derived from summing (M + I + E1 +E2) scores from the 5 segments examined, the maximum score is 75. (TIF) [file pone.0239295.s002.tif]

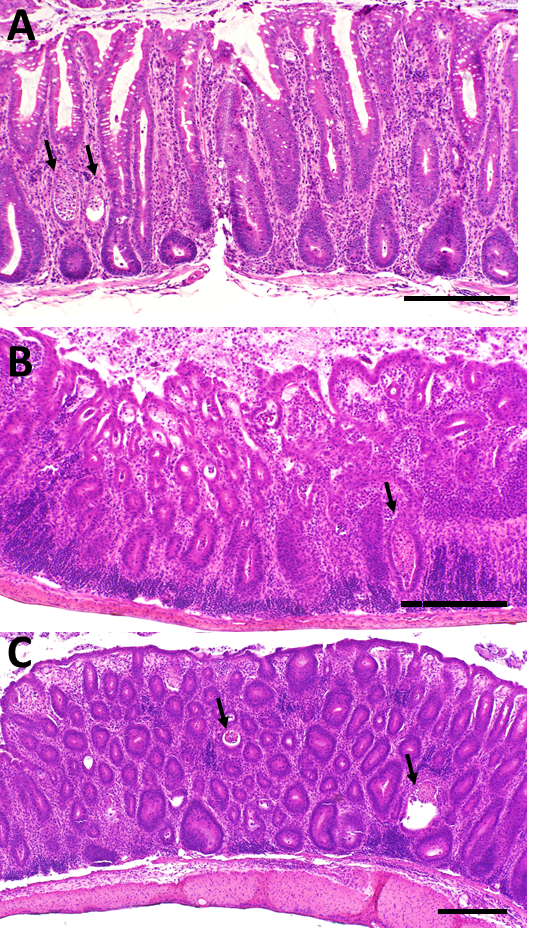

Supplement: S3 Fig — T/I mice generally developed moderate to severe mucosal inflammation involving cecum to rectum (“pan-colitis”) soon after weaning, as described in [20]. The representative fields shown are from the terminal colon/rectum (A), distal colon (B), and mid-colon (C). Marked epithelial hyperplasia is present and the lamina propria is packed with inflammatory cells. Representative crypt abscesses are indicated by arrows. Scale bar represents 250 μm in panels A and B and 500 μm in panel C. (TIF) [file pone.0239295.s003.tif]

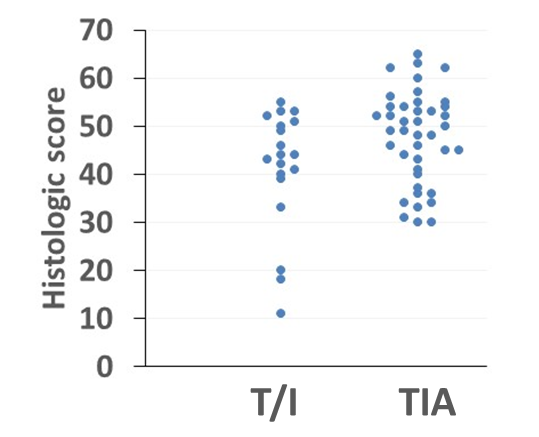

Supplement: S4 Fig — Graph shows histologic scores for T/I (n = 19) and TIA mice (n = 41) between the ages of 12 and 28 weeks who were euthanized for determination of neoplasia, either when they met humane endpoints or at the experimental endpoint of 28 weeks. Each point represents a single mouse. p = 0.06 (Student’s t-test). (TIF) [file pone.0239295.s004.tif]
